# Integrin α5β1 Activation by PHSRN Peptide Elicits Neuroprotection and Functional Recovery in Parkinson’s Disease Mice

**DOI:** 10.3390/antiox15070822

**Published:** 2026-06-30

**Authors:** Cheng-Chun Wu, Hao-Kuang Wang, Yu-Ting Su, Yu-Cheng Ho, Yuan-Chin Hsieh, Cheng-Loong Liang, Yung-Kuo Lee, Tian-Huei Chu, Yun-Shin Lin, Jui-Sheng Chen

**Affiliations:** 1 Graduate Institute of Medicine, College of Medicine, I-Shou University, Kaohsiung City 824005, Taiwan; chengchunwu@isu.edu.tw (C.-C.W.); ed101393@gmail.com (H.-K.W.); 2 School of Medicine, College of Medicine, I-Shou University, Kaohsiung City 824005, Taiwan; ycho@isu.edu.tw (Y.-C.H.); p0201@edah.org.tw (C.-L.L.); 3 Department of Neurosurgery, E-DA Hospital, I-Shou University, Kaohsiung City 824005, Taiwan; 4 Department of Obstetrics and Gynecology, Kaohsiung Chang Gung Memorial Hospital and Chang Gung University College of Medicine, Kaohsiung City 833401, Taiwan; kimyy9487@cgmh.org.tw; 5 Department of Occupational Therapy, College of Medicine, I-Shou University, Kaohsiung City 824005, Taiwan; hyc1014@isu.edu.tw; 6 Medical Laboratory, Medical Education and Research Center, Kaohsiung Armed Forces General Hospital, Kaohsiung City 802231, Taiwan; yungkuolee@gmail.com (Y.-K.L.); skbboyz0817@gmail.com (T.-H.C.); 7 Division of Experimental Surgery Center, Department of Surgery, Tri-Service General Hospital, National Defense Medical University, Taipei 114201, Taiwan; 8 School of Medicine, National Defense Medical University, Taipei 114201, Taiwan; 9 Department of Psychiatry, Kaohsiung Armed Forces General Hospital, Kaohsiung City 802231, Taiwan; mysing@gmail.com

**Keywords:** Parkinson’s disease, integrin α5β1, PHSRN peptide, dopaminergic neuroprotection, oxidative stress, NGF, BDNF, synaptic integrity

## Abstract

Parkinson’s disease (PD) is characterized by progressive dopaminergic neurodegeneration driven by oxidative stress, mitochondrial dysfunction, synaptic loss, and impaired neurotrophic signaling; however, the role of integrin α5β1 in neuronal vulnerability remains unclear. Here, the data show that rotenone-induced stress reduces integrin α5 expression in a dose- and time-dependent manner, leading to increased ROS accumulation, glutathione imbalance, synaptic degeneration, senescence-like β-gal activity, and apoptosis, whereas integrin α5 knockdown further exacerbates these deficits, supporting a protective role of α5β1. In contrast, treatment with the fibronectin-derived α5β1-activating peptide Ac-PHSRN-NH_2_ restores integrin signaling by engaging the FAK–PI3K–AKT/ERK cascade and NRF2-mediated antioxidant responses, thereby reducing oxidative stress, suppressing cell death, and improving redox homeostasis. Moreover, PHSRN enhances NGF and BDNF levels, preserves synaptic integrity, and promotes dopaminergic neuronal activity and dopamine release. Consistently, in MPTP-lesioned mice, PHSRN preserves nigral TH-positive neurons, reduces apoptosis, restores neurotrophic support, and improves motor function. Collectively, these findings identify integrin α5β1 as a critical protective axis and support PHSRN as a potential disease-modifying therapeutic strategy for PD.

## 1. Introduction

Parkinson’s disease (PD) is a progressive neurodegenerative disorder characterized by the selective loss of dopaminergic neurons in the substantia nigra pars compacta, leading to dopamine depletion and basal ganglia dysfunction. Clinically, this manifests as bradykinesia, resting tremor, rigidity, and postural instability, often followed by cognitive decline and autonomic dysfunction at later stages [1,2,3]. Affecting over 8.5 million people worldwide, PD shows a prevalence exceeding 1% among individuals over 65 and 2.5% in those over 80, reflecting its growing impact in aging populations. Current treatments—mainly dopamine replacement therapy and deep brain stimulation—provide symptomatic relief but fail to halt disease progression [4,5,6]. At the molecular level, misfolded α-synuclein aggregates into Lewy bodies, triggering oxidative stress, mitochondrial dysfunction, and neuroinflammation [7]. The accumulation of reactive oxygen species (ROS) promotes lipid peroxidation, DNA damage, and mitochondrial impairment, ultimately leading to neuronal death [8,9,10]. In parallel, deficits in neurotrophic factors such as BDNF, NGF, and IGF-1 further weaken neuronal survival, synaptic maintenance, and axonal transport, exacerbating dopaminergic degeneration [11,12].

Integrins are key mediators linking extracellular cues to intracellular signaling. These transmembrane receptors form focal adhesion complexes that regulate cell–ECM interactions, cytoskeletal organization, and downstream pathways. In the nervous system, integrins control neuronal adhesion, migration, and axonal outgrowth [13,14]. Among them, integrin α5β1—a fibronectin-binding receptor—plays a central role in neurodevelopment, neurite extension, and synaptogenesis, and may also influence neurotrophin signaling and expression [15,16,17,18]. Integrin α5β1 are expressed in TH-positive mesencephalic dopaminergic neuronal soma, growth cones, and varicosities, and their mRNA expression has also been detected in the substantia nigra and ventral tegmental area [19,20]. Their localization to growth cones and dynamic neuronal processes suggests a potential role for integrin α5β1 in dopaminergic neurite outgrowth, regeneration, and structural remodeling [21,22]. Upregulation of α5β1 in ischemic or damaged brain regions further suggests its involvement in repair and pathological remodeling [23,24].

The fibronectin-derived peptide PHSRN (Pro–His–Ser–Arg–Asn), located within the type III domain, enhances α5β1 binding through synergy with the RGD motif and modulates integrin-mediated adhesion, signaling, and cytoskeletal dynamics [13,25,26,27,28]. PHSRN has shown therapeutic potential in promoting epithelial repair [29,30], endothelial migration and angiogenesis [24,31], and neuronal adhesion and growth [24,32]. In stroke models, PHSRN administration reduced infarct volume, improved neurological outcomes, and enhanced angiogenesis, likely via the α5β1-dependent activation of FAK, Ras, and ERK signaling [24,32]. Despite these findings, the role of α5β1 integrin and its modulation by PHSRN in Parkinson’s disease remains largely unexplored.

In this study, we explored the contribution of integrin α5β1 signaling to Parkinson’s disease pathogenesis and evaluated the therapeutic efficacy of exogenous PHSRN peptide in preserving dopaminergic neuronal integrity and restoring motor function in a PD mouse model, highlighting integrin α5β1 as a potential target for neuroprotection.

## 2. Materials and Methods

### 2.1. Animal Model Induction

All procedures followed ARRIVE 2.0 guidelines (https://arriveguidelines.org; accessed on 1 May 2026) and were approved by the IACUCs of I-Shou University and E-Da Hospital (IACUC-ISU-109029, 113022; IACUC-EDAH-112033). Male C57BL/6J mice (8 weeks, 20–25 g; Lasco Biotechnology, Taipei, Taiwan) were housed under SPF conditions (12 h light/dark cycle, ad libitum food and water) and acclimated for 7 days before experiments. A total of 114 mice were randomly assigned to four groups based on prior power calculations and pilot data to ensure adequate tissue for behavioral, histological, and molecular analyses. Parkinsonian pathology was induced using a modified MPTP/probenecid protocol [33]: intraperitoneal MPTP (25 mg/kg/day) and probenecid (250 mg/kg) for 5 days. Normal saline was administered intraperitoneally as the vehicle control. After behavioral testing, mice were deeply anesthetized with Zoletil (40 mg/kg, i.p.) before transcardial perfusion. Brains were collected in PBS for protein extraction or fixed with paraformaldehyde for immunostaining. Only animals that remained healthy and recovered uneventfully were included. Exclusion criteria were surgical mortality and injection site complications. All assessments were performed blinded, with randomized cage positions and consistent testing schedules to minimize bias.

### 2.2. Drug Administration

The peptide Ac-Pro-His-Ser-Arg-Asn-NH_2_ (Ac-PHSRN-NH_2_) was synthesized according to previous reports describing its synergistic interaction with the Arg-Gly-Asp (RGD) motif in fibronectin and its roles in neuroprotection and tissue repair [24,34]. N-terminal acetylation and C-terminal amidation were included to improve chemical stability. Ac-PHSRN-NH_2_ was custom-synthesized by Mission Biotech (Taipei, Taiwan) with a purity ≥95% verified by reverse-phase HPLC. Peptide identity was confirmed by mass spectrometry and amino acid analysis. The lyophilized peptide was reconstituted in sterile saline and administered intraperitoneally (0.5 mg/kg) for 14 consecutive days following 5 days of MPTP + probenecid injection. Saline-treated animals served as controls.

### 2.3. Integrin α5 Knock Down In Vivo

Mice were anesthetized with isoflurane (4–5% for induction and 1.5–2.5% for maintenance in oxygen) and secured in a stereotaxic apparatus. A lentiviral vector carrying shRNA targeting mouse integrin α5-GFP (AS1010-sh262763), designed and purchased from the RNAi Core of the National Core Facility for Biopharmaceuticals at Academia Sinica (Taiwan), was bilaterally injected into the substantia nigra (coordinates: AP = −3.7 mm; ML = ±1.5 mm; DV = −3.9 mm) using a Hamilton syringe at a rate of 0.2 μL/min. Sham-operated mice received vehicle injections (HBSS) using the same stereotactic procedure. For the MPTP + integrin α5 knockdown group, stereotactic injection of a lentiviral vector expressing shRNA against integrin α5 tagged with GFP was performed three weeks prior to MPTP administration. Knockdown efficiency and viral infection were confirmed by GFP expression in the substantia nigra using immunofluorescence analysis. All surgeries were performed under aseptic conditions. Perioperative analgesia was provided using subcutaneous buprenorphine (0.05 mg/kg every 8–12 h for 48 h), in accordance with the 2020 AVMA Guidelines.

### 2.4. Cell Culture, Integrin α5 Knockdown, Drug Treatment, and SA-β-Gal Staining

N27 cells were seeded onto dishes precoated with poly-L-lysine (50 µg/mL; Sigma-Aldrich, St. Louis, MO, USA. Cat# P4707) and fibronectin (20 µg/mL; Sigma-Aldrich, Cat# F1141). Cells were maintained at 37 °C in 5% CO_2_ in complete medium supplemented with 10% fetal bovine serum (FBS; Sigma-Aldrich, Cat# F7524) and antibiotics. For integrin α5 knockdown, cells were transfected with 100 pmol integrin α5 siRNA (Invitrogen, Carlsbad, CA, USA. Cat# s68430) using Lipofectamine RNAiMAX (Invitrogen, Cat# 13778030) according to the manufacturer’s protocol. Cells were cultured for 3 days after transfection before chemical treatment. Twenty-four hours after knockdown, cells were then treated with rotenone (1–10 nM; Sigma-Aldrich, Cat# R8875) with or without PHSRN peptide (25 µM) for 24 h. Senescence-associated β-galactosidase activity was detected using a β-Galactosidase Staining Kit (Cell Signaling Technology, Danvers, MA, USA) according to the manufacturer’s instructions. SA-β-gal-positive cells were identified by blue cytoplasmic staining and quantified as the percentage of positive cells among total cells.

### 2.5. Gait Analysis

Gait analysis was performed 24 h after chemical administration following the previously described protocol [35]. C57BL/6J mice were transferred to the testing room and acclimated for 30 min before testing. The apparatus consisted of a 90 cm × 5 cm × 15 cm tunnel constructed from 0.3 mm foam board, with white paper strips placed on the floor to record footprints. The forepaws and hindpaws were coated with non-toxic, water-based paint (blue for forelimbs, red for hindlimbs). Mice were allowed to traverse the tunnel voluntarily. Stride length, base width, and total step count were measured manually using a ruler. All recordings and analyses were conducted in a blinded manner to minimize observer bias.

### 2.6. Immunofluorescence, TUNEL, and β-Galactosidase Staining

Immunofluorescence, TUNEL, and β-galactosidase staining were performed using standard protocols. Mice were deeply anesthetized and transcardially perfused with PBS followed by 4% paraformaldehyde (PFA; Sigma-Aldrich, St. Louis, MO, USA). Brains were post-fixed, cryoprotected in 30% sucrose, embedded in OCT (Sakura Finetek, Torrance, CA, USA), and coronally sectioned at 10 μm through the substantia nigra pars compacta (SNc). Sections were mounted on Superfrost Plus slides (Thermo Fisher Scientific, Waltham, MA, USA), blocked in 5% normal goat serum with 0.3% Triton X-100, and incubated overnight at 4 °C with primary antibodies against tyrosine hydroxylase (ab152, Abcam), PSD-95 (36233S, CST), NeuN (MAB377, Millipore, Burlington, MA, USA), and cleaved caspase-3 (9661S, CST). Alexa Fluor-conjugated secondary antibodies (Thermo Fisher Scientific) were applied, and nuclei were counterstained with DAPI. Apoptosis was assessed using the In Situ Cell Death Detection Kit (Millipore) according to the manufacturer’s instructions. Sections were coverslipped with Dako Fluorescence Mounting Medium (Dako, Glostrup, Denmark) and imaged using the ImageXpress Micro Confocal system (Molecular Devices, San Jose, CA, USA). Image acquisition and analysis were performed with MetaXpress software (v6.7) under standardized exposure and focus settings. DAPI was used for nuclear segmentation, and target signals (FITC or Texas Red) were quantified using the Cell Scoring module. All image analyses were conducted in a blinded manner.

### 2.7. Western Blot, ELISA, and Biochemical Assays

Protein lysates from the striatum and substantia nigra pars compacta (SNc) were prepared using a brain matrix and homogenized in RIPA buffer (Thermo Fisher #89900) containing protease and phosphatase inhibitors (Roche, Basel, Switzerland. #11873580001). After centrifugation at 12,000× *g* for 20 min at 4 °C, supernatants were collected, and protein concentrations were measured using a BCA kit (Thermo Fisher #23227). Equal protein amounts (30–50 µg) were separated by SDS-PAGE, transferred to PVDF membranes (Millipore #IPVH00010), blocked with 5% milk, and incubated overnight at 4 °C with primary antibodies against integrin α5 (GeneTex, Irvine, CA. GTX130705), GAPDH (Proteintech, Rosemont, IL. 60004-I-1G), PSD-95 (CST 36233S), cleaved caspase-3 (CST 9661S), p-FAK (CST 3283S), FAK (GeneTex GTX100764), PI3K (GeneTex GTX111068), p-AKT (CST 9271S), AKT (GeneTex GTX121937), p-ERK (Invitrogen 44-680G), ERK (Invitrogen 61-7400), and caspase-8 (GeneTex GTX110723). HRP-conjugated secondary antibodies (CST #7074/7076) and ECL substrate (Thermo Fisher #32106) were used for detection. Band intensity was quantified with ImageJ (ij154-osx-arm-java13). ELISA and biochemical assays were performed on fresh lysates per manufacturers’ instructions using kits for integrin α5 (MBS2887565), TUNEL (C10618), NGF (EM9RB), BDNF (DBNT00), LDH (AB65391), ROS (ab83464), β-galactosidase (C10850), and GSH (ab138881). Absorbance or fluorescence was measured in triplicate using a SpectraMax iD3 reader (Molecular Devices, San Jose, CA, USA).

### 2.8. Dopamine Level Measurement

Mouse brains were rapidly removed after sacrifice and sectioned using a brain slicer. Coronal midbrain sections corresponding approximately to bregma −2.80 to −3.80 mm were collected. Bilateral lower midbrain tissue blocks containing the substantia nigra region were dissected, homogenized, and processed for dopamine measurement. Dopamine levels were quantified using a dopamine ELISA kit (Novus Biologicals, Centennial, CO, USA. Cat# NBP2-67270) according to the manufacturer’s instructions. Dopamine concentrations were calculated from the standard curve.

### 2.9. Ex Vivo Brain Slice Electrophysiological Recording of Dopaminergic Neurons

Whole-cell patch-clamp recordings were performed on acute midbrain slices from 8-week-old male C57BL/6 mice following behavioral testing. Mice were deeply anesthetized with isoflurane and perfused with ice-cold, oxygenated dissection solution (in mM: 110 choline, 25 NaHCO_3_, 25 D-glucose, 11.6 sodium ascorbate, 7 MgSO_4_, 3.1 sodium pyruvate, 2.5 KCl, 1.25 NaH_2_PO_4_, and 0.5 CaCl_2_). Coronal brain slices (250 μm) containing the substantia nigra pars compacta (SNc) were prepared using a vibratome (5100 mz, Campden Instruments, Leicestershire, UK) and incubated in artificial cerebrospinal fluid (aCSF: 117 NaCl, 4.5 KCl, 2.5 CaCl_2_, 1.2 MgCl_2_, 1.2 NaH_2_PO_4_, 25 NaHCO_3_, and 11.4 dextrose) at room temperature for at least 1 hour prior to recording. For recordings, each slice was transferred to a chamber mounted on a fixed-stage upright IR-DIC microscope (BX51WI, Olympus, Tokyo, Japan) equipped with a 40× water-immersion objective and continuously superfused with aCSF at a flow rate of 3 mL/min. Patch electrodes (3–5 MΩ) were filled with internal solution (in mM: 125 potassium gluconate, 5 KCl, 0.5 CaCl_2_, 5 BAPTA, 10 HEPES, 5 MgATP, and 0.33 GTP-Tris; pH 7.3, 280 mOsm/L). Neurons were identified as dopaminergic if they exhibited an Ih current in response to hyperpolarizing voltage steps. Step currents (300 ms, 0–200 pA in 10 pA increments) were injected to assess neuronal firing frequency. Signals were acquired and sampled at 5–10 kHz using pClamp 11.1 via a MultiClamp 700B amplifier and a Digidata 1550B A/D converter, and analyzed with Clampfit 11.1.

### 2.10. Data Analysis and Statistics

All datasets were tested for normality and presented as mean ± SEM. Statistical comparisons were conducted using one-way ANOVA with post hoc Tukey’s test for multiple-group comparisons. A *p*-value < 0.05 was considered statistically significant.

## 3. Results

### 3.1. Integrin α5 Knockdown Exacerbates Gait Performance in Parkinsonian Mice

**Figure 1:**
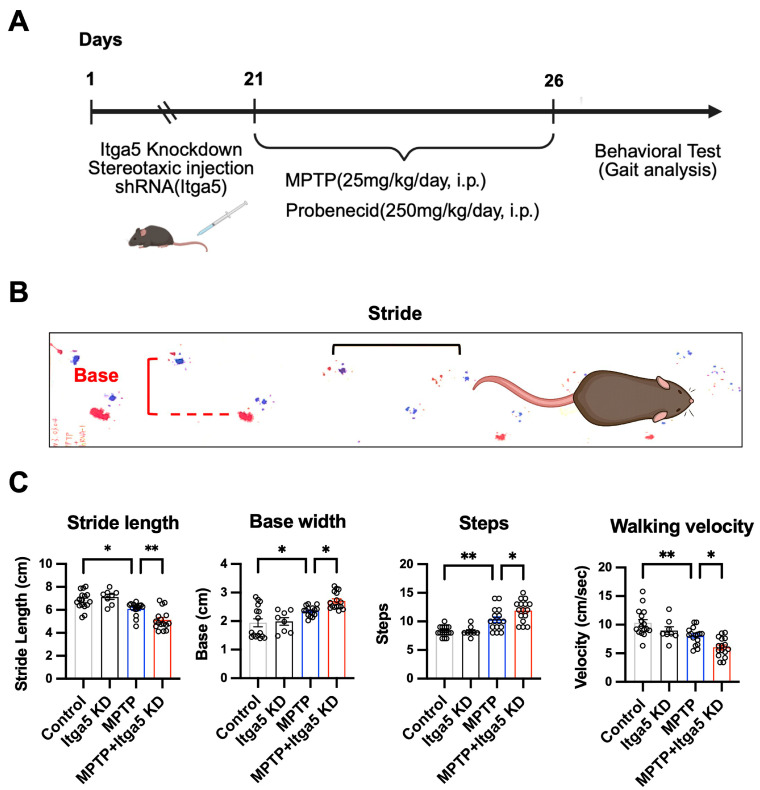
**Integrin α5 knockdown exacerbates MPTP-induced gait abnormalities in mice.** (**A**) Experimental timeline illustrating the stereotaxic delivery of lentiviral shRNA targeting *Itga5* into the substantia nigra on Day 1, followed by systemic administration of MPTP (25 mg/kg/day, i.p.) and probenecid (250 mg/kg/day, i.p.) for 5 consecutive days starting on Day 21. Behavioral assessment via gait analysis was conducted on Day 26. (**B**) Representative footprint patterns from hindlimbs (red) and forelimbs (blue), showing stride length and base width used for gait quantification. (**C**) Quantitative analysis of gait parameters revealed that MPTP-treated mice exhibited significantly reduced stride length and walking velocity, along with increased base width and number of steps compared to controls. These impairments were further exacerbated in the MPTP + *Itga5* KD group, indicating worsened motor coordination and postural instability. Control: N = 16; *Itga5* KD: N = 8; MPTP: N = 16; MPTP + *Itga5* KD: N = 16. Data are presented as mean ± SEM. * *p* < 0.05, ** *p* < 0.01 by one-way ANOVA with Tukey’s post hoc test.

To investigate the role of integrin α5β1 in Parkinson’s disease (PD) pathogenesis, a PD mouse model was established using MPTP administration. Lentiviral-mediated knockdown of integrin α5 (Itga5 KD) was performed three weeks prior to MPTP induction via stereotactic injection into the substantia nigra pars compacta (SNc) (Figure 1A). Knockdown efficiency was confirmed by immunofluorescence and Western blot analyses (Figure S1A,B). Gait analysis was subsequently conducted in control, MPTP, Itga5 KD, and MPTP + Itga5 KD groups. Footprints were collected to quantify hindlimb stride length, base width, step number, and walking velocity (Figure 1B). In the quantifications, MPTP-treated mice exhibited significant motor deficits compared with controls, characterized by reduced stride length, increased base width, higher step frequency, and slower walking velocity (*p* < 0.01). These impairments were further aggravated in the MPTP + Itga5 KD group, which showed the most pronounced shortening of stride length, widening of gait base, increased number of steps, and decreased walking speed relative to the MPTP-only group (*p* < 0.05 to *p* < 0.01; Figure 1C). These findings indicate that integrin α5β1 deficiency exacerbates MPTP-induced locomotor dysfunction, suggesting its role as a critical role in pathogenesis of Parkinson’s disease.

### 3.2. Integrin α5β1 Modulates Dopaminergic Neuron Susceptibility to Rotenone Toxicity

**Figure 2:**
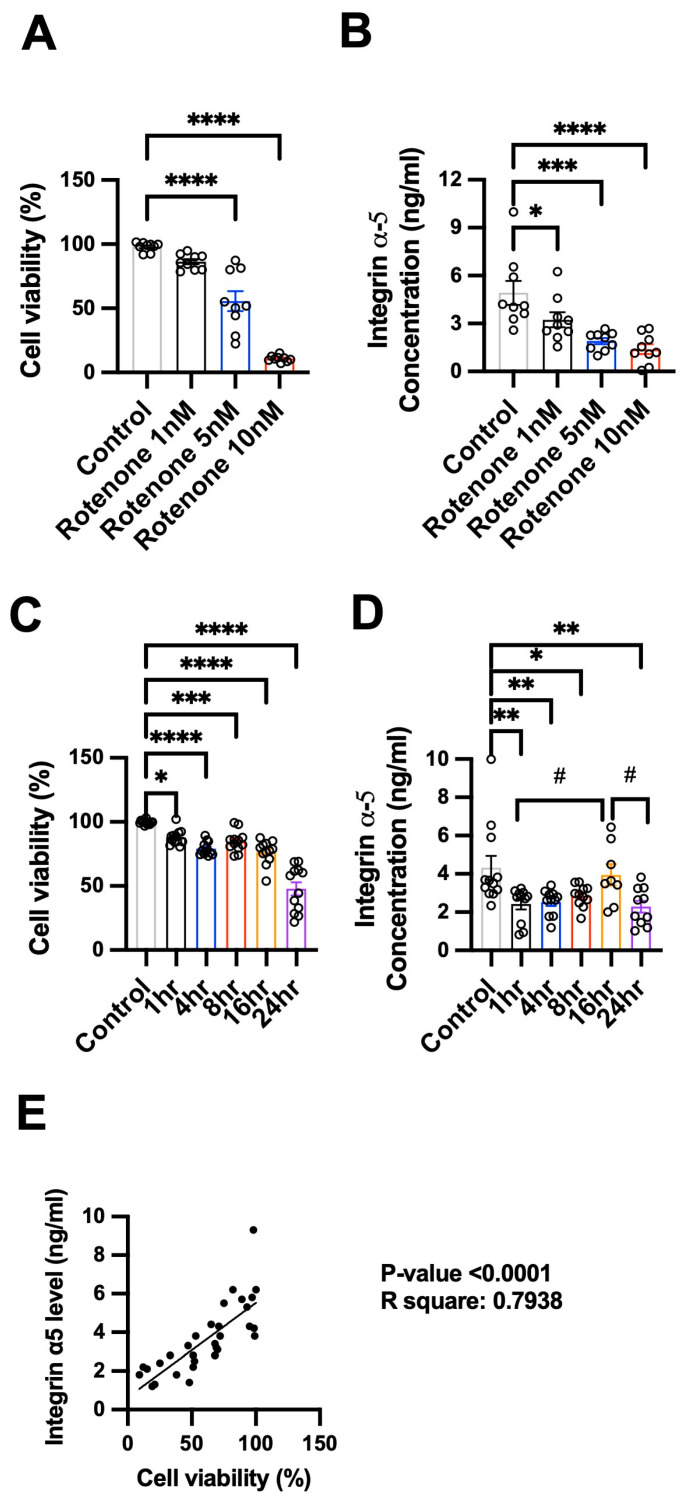
**Integrin α5β1 Expression Positively Correlates with Dopaminergic Neuron Viability under Rotenone Exposure.** (**A**) N27 neurons were seeded on poly-L-lysine (50 µg/mL) and fibronectin (20 µg/mL)-coated dishes for 24 h. Dose-dependent effects of rotenone on N27 dopaminergic neuron viability were measured by AlamarBlue assay. Cells were exposed to rotenone at 1, 5, or 10 nM for 1 h, resulting in a concentration-dependent reduction in viability. N = 9 independent experiments each group. **** *p* < 0.0001 by one-way ANOVA with Tukey’s post hoc test. (**B**) Integrin α5 protein expression measured by ELISA, decreased progressively with increasing rotenone concentrations. N = 9 independent experiments each group. * *p* < 0.05, *** *p* < 0.001 to **** *p* < 0.0001 by one-way ANOVA with Tukey’s post hoc test. (**C**) Time-course effects of 1 nM rotenone on cell viability. A significant decline in viability was observed from 1 h onward, reaching the lowest levels at 24 h. N = 8–12 independent experiments each group. * *p* < 0.05, *** *p* < 0.001 to **** *p* < 0.0001 by one-way ANOVA with Tukey’s post hoc test. (**D**) Time-dependent changes in integrin α5 expression following 1 nM rotenone treatment. Expression was significantly reduced at 1, 4, 8, and 24 h, with a transient increase observed at 16 h. N = 8–12 independent experiments each group. *, # *p* < 0.05 to ** *p* < 0.001 by one-way ANOVA with Tukey’s post hoc test. (**E**) Correlation between integrin α5 expression and cell viability in rotenone-treated N27 dopaminergic neurons. Data were analyzed using linear regression, revealing a significant positive correlation (R^2^ = 0.7938, *p* < 0.0001). Each dot represents data from an independent replicate.

To elucidate the involvement of integrin α5β1 in PD pathogenesis, we assessed cell viability and integrin α5 expression in N27 dopaminergic neurons following rotenone exposure. In the dose-dependent experiment, increasing concentrations of rotenone (1, 5, and 10 nM) led to a significant reduction in cell viability in a concentration-dependent manner after exposure to rotenone for 1 h (Figure 2A, *p* < 0.0001). Correspondingly, enzyme-linked immunosorbent assay (ELISA) analysis revealed a marked decrease in integrin α5 protein levels with increasing rotenone concentrations (Figure 2B, *p* < 0.05 to *p* < 0.0001), suggesting a link between rotenone-induced neurotoxicity and integrin α5 downregulation. In addition, in the time-course experiment using 1 nM rotenone, cell viability declined progressively over time, with significant reductions observed from 1 h onward and peaking at 24 h (Figure 2C, *p* < 0.05 to *p* < 0.0001). Similarly, integrin α5 expression decreased in a time-dependent manner, with significant reductions at 1, 4, 8, and 24 h (Figure 2D, *p* < 0.05 to *p* < 0.01), though a transient increase was observed at 16 h. Furthermore, to determine the relationship between integrin α5 expression and neuronal viability, correlation analysis was performed using data from rotenone-treated N27 dopaminergic cells. A significant positive correlation was observed between integrin α5 protein levels and cell viability (R^2^ = 0.7938, *p* < 0.0001; Figure 2D), indicating that higher integrin α5 expression was associated with greater neuronal survival. These results suggest that integrin α5 may play a neuroprotective role in response to rotenone-induced toxicity and support its potential involvement in dopaminergic neuronal vulnerability in PD models.

### 3.3. Integrin α5 Knockdown Exacerbates Rotenone-Induced Dopaminergic and Synaptic Injury In Vitro

**Figure 3:**
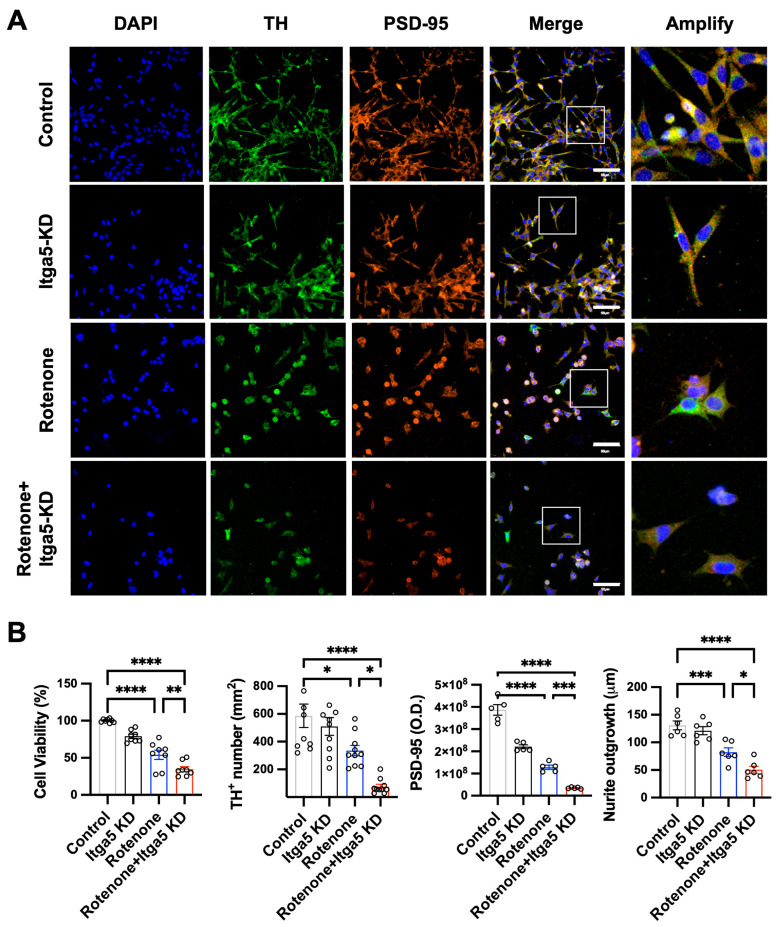
**Integrin α5 knockdown exacerbates rotenone-induced degeneration of dopaminergic neurons.** (**A**) Representative immunofluorescence images of differentiated neuronal cultures showing nuclei (DAPI, blue), dopaminergic neurons (TH, green), and postsynaptic density protein PSD-95 (red). Control and Itga5 knockdown alone exhibited preserved dopaminergic morphology 24 h after experimental insult. Rotenone treatment (5 nM rotenone for 1 h) induced marked neuronal degeneration, which was further aggravated in *Itga5* knockdown + rotenone cultures. Amplified panels highlight reductions in TH-positive cell morphology and PSD-95 synaptic puncta. Scale bars = 20 μm. (**B**) Quantification of cell viability, TH-positive neuron density, PSD-95 optical density, and neurite outgrowth length across indicated experimental groups. Rotenone significantly impaired all measurements compared to control, and these impairments were intensified by Itga5 knockdown. Data are mean ± SEM from ≥ 5 independent experiments. Statistical significance determined by one-way ANOVA with post hoc multiple comparisons: * *p* < 0.05, ** *p* < 0.01, *** *p* < 0.001, **** *p* < 0.0001.

To investigate the role of integrin α5 in dopaminergic vulnerability to neurotoxicity, we knocked down integrin α5 (Itga5 KD) in N27 dopaminergic neurons and assessed dopaminergic and synaptic markers following rotenone exposure. For detail on the knock down efficiency in vitro please see Figure S1B. As shown in Figure 3A, Itga5 knockdown alone did not markedly alter TH expression or neuronal morphology compared with control cultures. In contrast, rotenone exposure led to a substantial reduction in TH-positive neurons, diminished PSD-95 signal intensity, and shortened neuritic processes. Notably, the combined condition of Itga5 knockdown and rotenone resulted in the most severe degeneration, characterized by profound loss of TH-immunoreactive neurons, marked reduction in PSD-95 puncta, and pronounced neurite atrophy. Quantitative analysis confirmed these observations. Rotenone significantly reduced cell viability, TH-positive neuron counts, PSD-95 expression, and neurite outgrowth (*p* < 0.05 to *p* < 0.0001); these deficits were further exacerbated by Itga5 knockdown (Figure 3B, *p* < 0.05 to *p* < 0.001). Together, these results indicate that integrin α5 is required to maintain dopaminergic neuronal integrity and synaptic structure, and that loss of Itga5 sensitizes neurons to rotenone–induced degeneration.

### 3.4. PHSRN Attenuates Dopaminergic Neuronal Loss and Apoptosis in an MPTP-Induced PD Mouse

**Figure 4:**
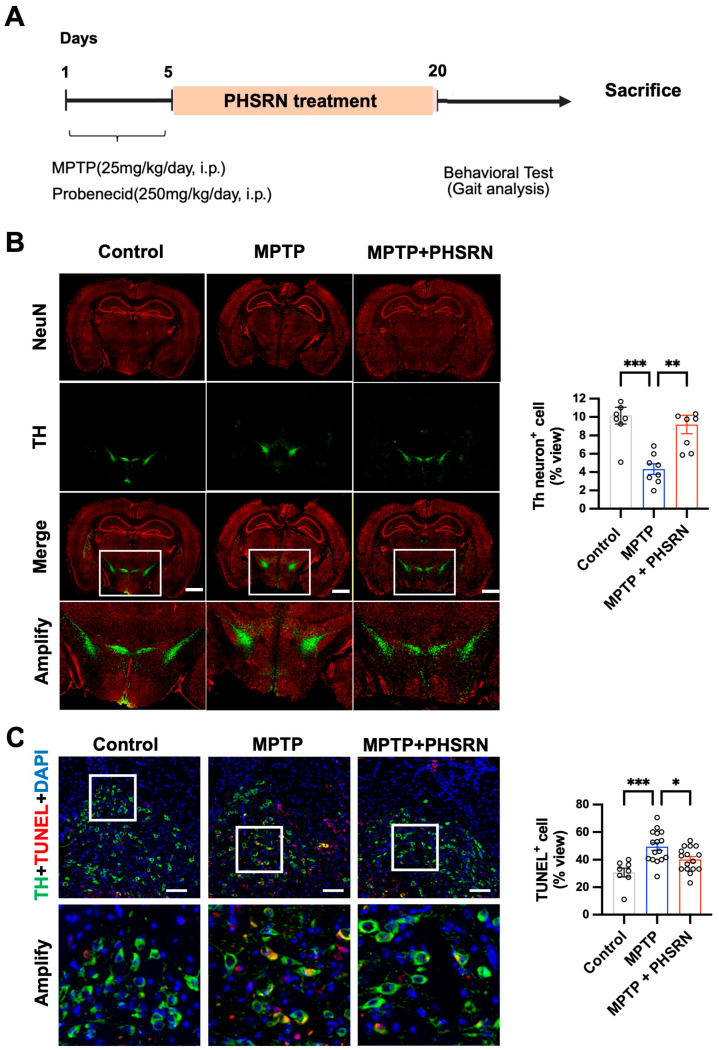
**PHSRN protects nigral dopaminergic neurons from MPTP-induced degeneration and apoptosis in vivo.** (**A**) Experimental timeline. Mice received MPTP (25 mg/kg/day, i.p.) and probenecid (250 mg/kg/day, i.p.) for 5 days. PHSRN was administered starting on day 6 and continued until sacrifice for 14 consecutive days. Behavioral assessment (gait analysis) was performed prior to sacrifice. (**B**) Representative immunofluorescence images of NeuN (red) and TH (green) in the substantia nigra from control, MPTP, and MPTP + PHSRN groups. Amplified views highlight the SNpc. Quantification shows a significant reduction in TH-positive neurons following MPTP, which was significantly preserved by PHSRN. Scale bars: 1 mm. N = 8 per group. (**C**) Representative images of TUNEL (red), TH (green), and DAPI (blue) labeling in the SNpc. Amplified views highlight apoptotic neurons. Quantification shows marked increases in TUNEL-positive neurons after MPTP, which were significantly attenuated by PHSRN treatment. Scale bars: 100 μm. N = 8–16 per group. Data are mean ± SEM; * *p* < 0.05, ** *p* < 0.01, *** *p* < 0.001 by one-way ANOVA with Tukey’s post hoc test.

Having established a neuroprotective role for integrin α5 under rotenone-induced stress, we next evaluated the therapeutic efficacy of PHSRN in an MPTP-induced Parkinson’s disease mouse model. Mice received intraperitoneal PHSRN injections (0.5 mg/kg/day) for two weeks following a 5-day course of MPTP/probenecid administration (Figure 4A). Dopaminergic neuron integrity was assessed by TH immunofluorescence in the substantia nigra pars compacta (SNc) (Figure 4B). As expected, MPTP treatment led to a substantial reduction in TH-positive neurons compared with controls (*p* < 0.001). PHSRN administration significantly restored TH immunoreactivity in the SNc, as reflected by an increase in TH-positive neuronal area (*p* < 0.01), indicating robust preservation of dopaminergic neurons. In addition, to further examine how PHSRN mitigates dopaminergic neuronal loss, TUNEL staining was performed in combination with TH immunostaining (Figure 4C). MPTP administration markedly increased TUNEL-positive cells within the TH-positive population (*p* < 0.001), consistent with enhanced apoptotic signaling. PHSRN treatment significantly reduced TUNEL-positive counts relative to the MPTP group (*p* < 0.05), demonstrating a strong anti-apoptotic effect. Together, these data show that PHSRN confers in vivo neuroprotection by preserving dopaminergic neuronal populations and attenuating apoptosis, supporting its therapeutic potential for Parkinson’s disease.

### 3.5. PHSRN Treatment Restores Neurotrophic Factor Levels and Reduces Neuronal Injury Markers in MPTP-Induced PD Mice

**Figure 5:**
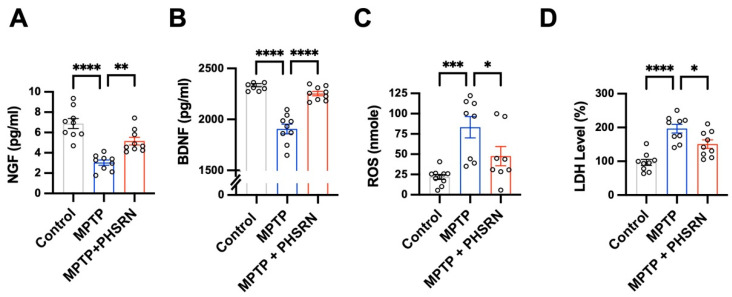
**PHSRN treatment restores neurotrophic factor levels and reduces neuronal injury markers in MPTP-induced Parkinson’s disease mice.** Enzyme-linked immunosorbent assay (ELISA) revealed that MPTP administration markedly reduced neurotrophin concentrations, including (**A**) NGF and (**B**) BDNF, compared with controls. PHSRN treatment significantly restored NGF levels (** *p* < 0.01) and partially rescued BDNF expression (**** *p* < 0.0001), indicating improved trophic support. (**C**) Reactive oxygen species (ROS) levels were significantly increased in MPTP-treated mice (*** *p* < 0.001), while PHSRN reduced ROS production to near control levels (* *p* < 0.05). (**D**) Lactate dehydrogenase (LDH), a marker of membrane damage and cytotoxicity, was elevated following MPTP exposure and was partially attenuated by PHSRN treatment (* *p* < 0.05 to **** *p* < 0.0001). N = 8–10 per group. Data are presented as mean ± SEM; significance was determined by one-way ANOVA with Tukey’s post hoc tests.

Neurotrophins and oxidative stress are well established as key contributors to PD pathogenesis [36,37]. To elucidate the mechanisms through which PHSRN preserves dopaminergic neurons, we quantified neurotrophic factors alongside oxidative and cellular injury markers in the MPTP-induced PD mouse model. MPTP administration significantly reduced NGF and BDNF levels compared with controls (*p* < 0.0001), whereas PHSRN treatment effectively restored NGF expression (Figure 5A, *p* < 0.01) and restored BDNF levels (Figure 5B, *p* < 0.0001), indicating enhanced neurotrophic support. In parallel, MPTP markedly elevated ROS production and LDH release (*p* < 0.001 to *p* < 0.0001), reflecting increased oxidative stress and membrane injury. PHSRN significantly reduced ROS levels (Figure 5C, *p* < 0.05) and attenuated LDH elevation (Figure 5D, *p* < 0.05), demonstrating mitigation of MPTP-induced cytotoxicity. Together, these findings indicate that PHSRN exerts neuroprotective effect through enhancement of neurotrophic signaling and reduction in oxidative and cellular injury.

### 3.6. PHSRN Attenuates Rotenone-Induced Senescence and Apoptosis in N27 Dopaminergic Neurons via an Integrin α5-Dependent Mechanism

**Figure 6:**
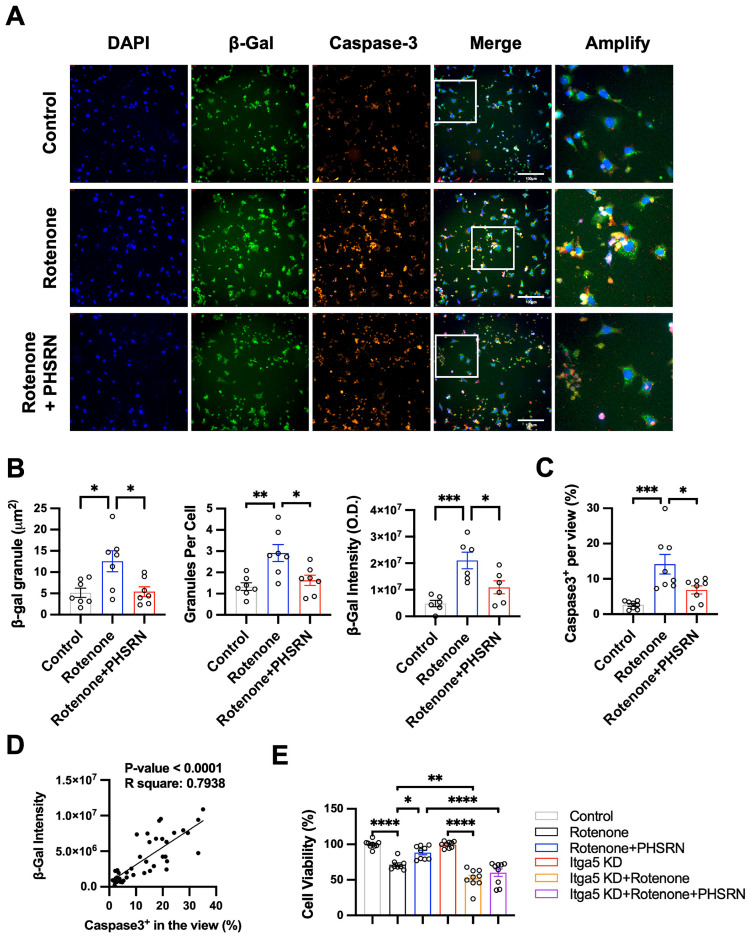
**PHSRN attenuates rotenone-induced degeneration and apoptosis in N27 dopaminergic neurons through an integrin α5-dependent mechanism.** (**A**) Representative immunofluorescence images showing β-galactosidase (β-Gal) staining and cleaved caspase-3 in N27 dopaminergic cells under control, rotenone, and rotenone + PHSRN conditions. In vitro experiments were performed by treating cells with 5 nM rotenone for 1 h, followed by PHSRN peptide treatment for 24 h (25 µM). DAPI, β-Gal, and TH immunofluorescence signals are shown in blue, green, and red, respectively. Scale bar, 100 μm. (**B**,**C**) Quantification demonstrated that rotenone significantly increased β-Gal granule area, density, intensity, and the proportion of cleaved caspase-3-positive cells, all of which were markedly reduced by PHSRN treatment. N = 4–8 independent experiments * *p* < 0.05, ** *p* < 0.01, *** *p* < 0.001 by one-way ANOVA with Tukey’s post hoc test. (**D**) The correlation analysis showed that β-Gal activity correlated positively with cleaved caspase-3-positive cell density, linking rotenone-induced senescence to apoptosis. PHSRN attenuated β-Gal activity and concomitantly reduced apoptotic cell death. (**E**) Cell viability measured by AlamarBlue assays revealed that rotenone exposure caused significant loss of viability (**** *p* < 0.0001 vs. control), partially rescued by PHSRN. Itga5 KD decreased baseline viability and exacerbated rotenone-induced toxicity, while abolishing PHSRN-mediated protection, indicating that PHSRN’s neuroprotective effects require integrin α5. N = 8 independent experiments. * *p* < 0.05, ** *p* < 0.01, **** *p* < 0.0001 by one-way ANOVA with Tukey’s post hoc test. Data are expressed as mean ± SEM.

The activity of β-galactosidase (β-Gal) is a widely used marker of cellular senescence under chronic stress [38]. In neurodegenerative conditions such as Parkinson’s and Alzheimer’s disease, neurons and glia exposed to oxidative stress, mitochondrial dysfunction, or DNA damage may enter a senescence-like state, characterized by ROS accumulation, DNA damage, and a close association with neuronal apoptosis and degeneration [39]. To determine whether rotenone-induced oxidative stress elicits a degenerative response in dopaminergic neurons, we performed β-Gal activity staining and cleaved caspase-3 immunostaining in the N27 dopaminergic cell line. Our data showed that rotenone treatment significantly increased β-Gal expression and cleaved caspase-3 immunoreactivity compared to control cells (Figure 6A). Quantification confirmed a robust upregulation of β-Gal intensity (Figure 6B, *p* < 0.05 to *p* < 0.001 vs. control) and a significant increase in cleaved caspase-3-positive cells (Figure 6C, *p* < 0.001 vs. control). Treatment with PHSRN markedly mitigated both β-Gal signal and cleaved caspase-3 activation (*p* < 0.05 vs. rotenone alone), indicating that PHSRN suppresses both cell stress and apoptosis in this oxidative injury model (Figure 6B,C). Correlation analysis across sampled fields revealed a significant positive relationship between β-Gal intensity and cleaved caspase-3-positive cell density (Figure 6D; *R*^2^ = 0.7938, *p* < 0.0001), suggesting that rotenone-induced stress is tightly linked to apoptotic commitment. Notably, PHSRN treatment attenuated β-Gal activity, thereby reducing apoptosis in dopaminergic neurons.

We next assessed cell viability under conditions of integrin α5 knockdown (Itga5 KD) to determine whether the neuroprotective effect of PHSRN is integrin-dependent. The data showed rotenone significantly reduced cell viability (Figure 6E; *p* < 0.0001 vs. control), which was partially rescued by PHSRN (*p* < 0.05 vs. rotenone). However, Itga5 knockdown further exacerbated rotenone toxicity (*p* < 0.01 vs. rotenone). Notably, in the context of Itga5 deficiency, PHSRN failed to restore cell viability, indicating that the neuroprotective effect of PHSRN is mediated through integrin α5. Together, these data indicate that rotenone drives a stress-apoptosis cascade in dopaminergic neurons and that PHSRN suppresses this cascade through an integrin α5-dependent mechanism.

### 3.7. PHSRN Protects Dopaminergic Neurons by Preserving Glutathione Redox Status and NRF2 Signaling

**Figure 7:**
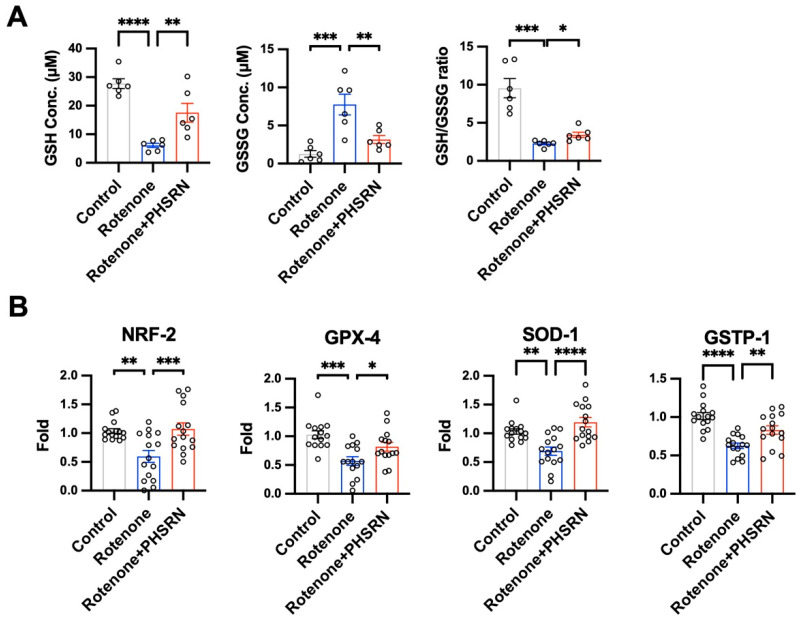
**PHSRN restores glutathione homeostasis and NRF2-mediated antioxidant gene expression in rotenone-treated N27 dopaminergic neurons.** (**A**) Biochemical analysis of midbrain tissue showed that rotenone administration significantly decreased reduced glutathione (GSH) levels and elevated oxidized glutathione (GSSG), resulting in a diminished GSH/GSSG ratio, consistent with oxidative stress (* *p* < 0.05 to *** *p* < 0.001). PHSRN treatment restored GSH concentrations, reduced GSSG accumulation, and normalized the GSH/GSSG ratio (* *p* < 0.05). In vitro experiments were performed by treating cells with 5 nM rotenone for 1 h, followed by PHSRN peptide treatment for 24 h (25 µM). N = 6 independent experiments. (**B**) Quantitative real-time PCR revealed that rotenone markedly suppressed the expression of antioxidant regulators Nrf2, Gpx4, Sod1, and Gstp1 (** *p* < 0.01 to **** *p* < 0.0001), which were significantly upregulated following PHSRN administration (* *p* < 0.05 to **** *p* < 0.0001). N = 15 independent experiments. Data are presented as mean ± SEM; statistical comparisons were performed using one-way ANOVA with Tukey’s post hoc analysis. Statistical significance determined by one-way ANOVA with post hoc multiple comparisons: * *p* < 0.05, ** *p* < 0.01, *** *p* < 0.001, **** *p* < 0.0001.

**To elucidate the antioxidant mechanisms contributing to the neuroprotective effects of PHSRN in vivo, we evaluated the glutathione redox system and the transcriptional profile of NRF2-dependent antioxidant genes in the N27 dopaminergic cell line.** Rotenone administration significantly decreased reduced glutathione (GSH) levels and increased oxidized glutathione (GSSG), resulting in a decreased GSH/GSSG ratio (*p* < 0.001 to *p* < 0.0001)—indicative of redox imbalance and oxidative stress (Figure 7A). PHSRN treatment effectively restored GSH levels, reduced GSSG accumulation, and ameliorated the GSH/GSSG ratio (*p* < 0.05 to *p* < 0.01), suggesting improved antioxidant capacity (Figure 7A). In addition, quantitative real-time PCR analysis of the midbrain tissue revealed that rotenone markedly suppressed the expression of *Nrf2*, *Gpx4*, *Sod1* and *Gstp1 (p* < 0.01 to *p* < 0.0001), key regulators of cellular redox defense (Figure 7B). PHSRN administration significantly reversed these transcriptional deficits, restoring antioxidant gene expression (Figure 7B, *(p* < 0.05 to *p* < 0.0001)). **These results indicate that PHSRN attenuates rotenone-induced oxidative stress by preserving glutathione redox balance and reactivating NRF2-mediated antioxidant pathways.**

### 3.8. PHSRN Targets FAK–PI3K–AKT/ERK Signaling to Mitigate Rotenone-Induced Neurodegeneration

**Figure 8:**
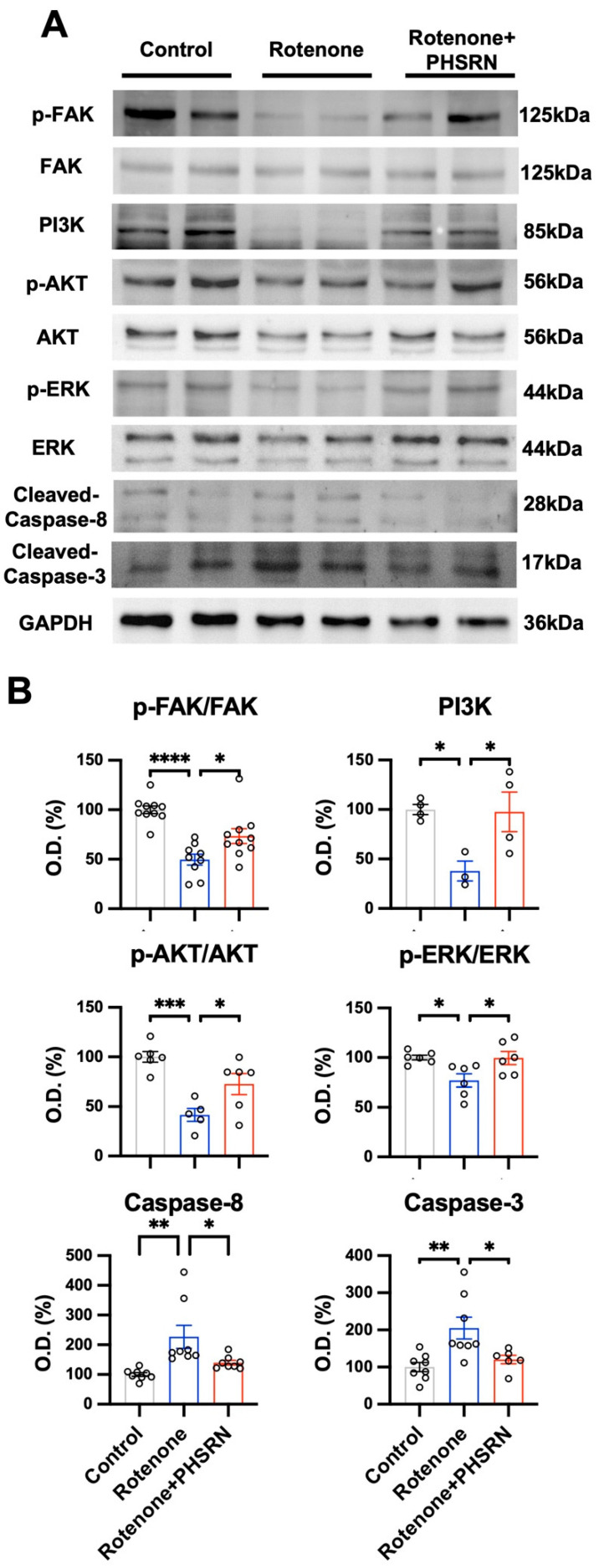
**PHSRN activates FAK–PI3K–AKT/ERK signaling and suppresses apoptotic pathways in rotenone-treated dopaminergic cells.** (**A**) Representative Western blots of N27 cell lysates showing phosphorylation of FAK, PI3K, AKT, and ERK, as well as cleaved caspase-8 and caspase-3, in control, rotenone-treated, and rotenone + PHSRN groups. GAPDH served as a loading control. In vitro experiments were performed by treating cells with 5 nM rotenone for 1 h, followed by PHSRN peptide treatment for 24 h (25 µM). (**B**) Quantification revealed that rotenone significantly reduced phosphorylation of FAK, AKT, ERK, and PI3K (* *p* < 0.05, *** *p* < 0.01, **** *p* < 0.0001), whereas PHSRN treatment restored the protein levels (* *p* < 0.05). Conversely, rotenone markedly increased cleaved caspase-3 and -8 (** *p* < 0.01), which were attenuated by PHSRN (* *p* < 0.05), indicating suppression of both intrinsic and extrinsic apoptotic cascades. N = 6–8 independent experiments. Data are presented as mean ± SEM, analyzed by one-way ANOVA with Tukey’s post hoc tests.

To investigate the intracellular signaling pathways involved in the neuroprotective effects of PHSRN in vitro, we performed Western blot analysis on cell lysates from control, rotenone-treated, and rotenone + PHSRN-treated N27 dopaminergic cells (Figure 8A). The data showed that rotenone exposure significantly reduced phosphorylation of FAK, PI3K, AKT, and ERK, indicating suppression of the integrin-linked survival signaling axis (*p* < 0.05 to *p* < 0.0001). Notably, treatment with PHSRN restored phosphorylation of these signaling proteins (*p* < 0.05), suggesting reactivation of FAK-mediated prosurvival pathways (Figure 8B). In parallel, we examined the expression of cleaved caspase-3 and cleaved caspase-8 as markers of apoptosis. Rotenone significantly increased the levels of both cleaved caspase-3 and -8, confirming the activation of apoptotic cascades. PHSRN treatment markedly attenuated this increase, indicating its anti-apoptotic effect (Figure 8B; *p* < 0.05). These findings demonstrate that PHSRN mitigates rotenone-induced neurotoxicity by restoring FAK–PI3K–AKT/ERK signaling and suppressing apoptotic pathway activation in the N27 dopaminergic neuron.

### 3.9. PHSRN Improves Motor Function, Dopamine Levels, Neuronal Excitability, and Synaptic Integrity in MPTP-Induced PD Mice

**Figure 9:**
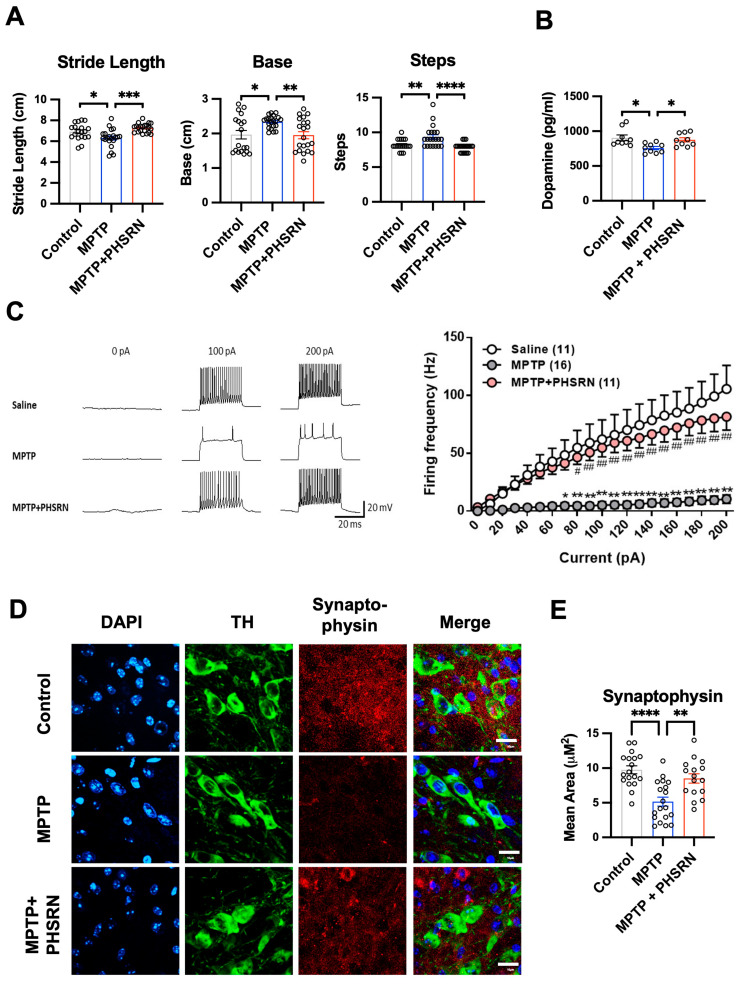
**PHSRN Reverses Motor Deficits and Restores Dopamine, Synaptic Density, and Neuronal Firing in MPTP-induced PD mice.** (**A**) Footprint-based gait analysis revealed reduced stride length, narrowed base width, and increased step count in MPTP-treated mice (* *p* < 0.05, ** *p* < 0.01, *** *p* < 0.001, **** *p* < 0.0001), all significantly improved by PHSRN (* *p* < 0.05 to **** *p* < 0.0001). N = 19–21 per group. (**B**) SNc dopamine concentrations were decreased by MPTP and partially restored with PHSRN (* *p* < 0.05). N = 9 per group. (**C**) Whole-cell patch-clamp recordings showed reduced firing frequency of dopaminergic neurons in MPTP-treated mice (* *p* < 0.05 to ** *p* < 0.01), which was significantly restored by PHSRN across current injections (# *p* < 0.05 to ## *p* < 0.01). N = 11–16 per group. (**D**,**E**) Representative immunofluorescent images of TH and synaptophysin staining in the SNc, with quantification showing reduced synaptic area in MPTP mice (**** *p* < 0.0001) and preservation with PHSRN treatment (** *p* < 0.01). DAPI, TH, and synaptophysin immunofluorescence signals are shown in blue, green, and red, respectively. Bar: 25 μm. Data are presented as mean ± SEM; significance determined by one-way or two-way ANOVA with Tukey’s post hoc tests.

To determine the functional benefits of PHSRN, we first assessed locomotor performance using footprint-based gait analysis. MPTP administration induced characteristic Parkinsonian gait deficits, including reduced stride length, narrowed base width, and an increased number of steps required to move across a fixed distance (Figure 9A, *p* < 0.05 to *p* < 0.01 vs. control). PHSRN treatment significantly improved all three parameters, restoring stride length, increasing base width, and reducing step count (Figure 9A, *p* < 0.05 to *p* < 0.0001 vs. MPTP). Next, dopamine concentrations in tissue samples from the dissected SNc region were measured, showing a marked decrease after MPTP exposure (Figure 9B, *p* < 0.05 vs. control), which was significantly attenuated by PHSRN (*p* < 0.05 vs. MPTP). Electrophysiological recordings further demonstrated impaired intrinsic firing frequency in dopaminergic neurons of MPTP mice across current steps (Figure 9C, *p* < 0.05 to *p* < 0.01 vs. Control + Saline), whereas PHSRN treatment restored neuronal excitability toward baseline levels (*p* < 0.05 to *p* < 0.01 vs. MPTP). Finally, immunofluorescence staining revealed that MPTP markedly reduced synaptophysin-positive presynaptic area and disrupted TH-positive dopaminergic morphology in the SNc (Figure 9D,E, *p* < 0.0001 vs. Control + Saline), while PHSRN preserved synaptic density and neuronal architecture (*p* < 0.0001 vs. MPTP). Together, these findings demonstrate that PHSRN ameliorates motor behavior, dopamine signaling, neuronal excitability, and synaptic integrity in a PD mouse model.

## 4. Discussion

Our results identify integrin α5β1 as a key neuroprotective regulator in dopaminergic neurons and demonstrate that Ac-PHSRN-NH_2_ activation of this receptor confers robust protection in experimental Parkinson’s disease. Systemic PHSRN treatment significantly improved motor performance in MPTP-lesioned mice, preserved nigral TH^+^ neurons, and reduced TUNEL-positive apoptotic profiles. PHSRN also prevented striatal dopamine loss and restored intrinsic firing activity of surviving SNc neurons. These effects were accompanied by preserved synaptic integrity and dendritic structure, reduced oxidative stress and LDH release, and partial restoration of NGF and BDNF levels. Together, these findings indicate that α5β1 activation by PHSRN effectively maintains dopaminergic neuron viability and function by preventing degeneration across behavioral, biochemical, and synaptic domains.

Regarding the use of different Parkinsonian toxins in the in vivo and in vitro experiments, MPTP and rotenone were used because each toxin is appropriate for a different experimental level. MPTP is a widely used in vivo Parkinsonian toxin that produces nigrostriatal dopaminergic degeneration and motor deficits after its conversion to MPP+ [33]. However, this metabolic conversion and tissue-level interaction are less suitable for a simplified cell culture system. Therefore, rotenone was used in vitro because it directly inhibits mitochondrial complex I and induces reproducible mitochondrial stress, oxidative injury, and dopaminergic neuronal damage in cultured cells [40,41]. Together, these complementary models allowed us to evaluate the protective effect of PHSRN at both the whole-animal and cellular mechanistic levels.

In a rotenone-induced oxidative stress model, PHSRN markedly attenuated the increase in SA–β-gal activity and cleaved caspase-3, indicating suppression of both senescence-like degeneration and apoptotic signaling. Consistent with these effects, PHSRN preserved dopaminergic cell viability under rotenone toxicity, in line with its pro-survival actions observed in vivo. Notably, rotenone exposure caused a dose- and time-dependent loss of integrin α5, paralleling declines in TH^+^ neuronal integrity, suggesting that dopaminergic neurons rely on α5β1 signaling to withstand rotenone toxicity. This aligns with previous work showing integrin-mediated survival signaling through FAK–AKT and MAPK pathways in neurons under oxidative and inflammatory challenge [42,43,44]. In our model, siRNA-mediated integrin α5 knockdown did not cause basal toxicity but significantly exacerbated rotenone-induced neurite retraction, synaptic PSD-95 loss, and TH^+^ neuron degeneration. These findings indicate that integrin α5β1 signaling serves as an endogenous protective axis that maintains dopaminergic structural and synaptic stability under stress. This neuron-centered mechanism is supported by the loss of PHSRN-mediated protection after Itga5 knockdown in dopaminergic cells. However, because integrin α5β1 may also be expressed in non-neuronal cells, including glial and vascular cells, additional indirect cellular contributions in vivo cannot be excluded and should be addressed in future cell type-specific studies.

We acknowledge that toxin-based Parkinsonian models do not fully recapitulate the progressive caudal-to-rostral propagation of α-synuclein pathology proposed in a subset of patients with Parkinson’s disease [45,46,47]. MPTP and rotenone models mainly reproduce mitochondrial complex I dysfunction, oxidative stress, and dopaminergic neuronal vulnerability [48,49], rather than α-synuclein aggregation or trans-synaptic spreading. This represents a limitation when interpreting the translational relevance of our findings. However, both α-synuclein-mediated pathology and toxin-induced Parkinsonian models ultimately converge on dopaminergic neuronal dysfunction and death. Therefore, our study was designed not to model α-synuclein propagation itself, but to test whether PHSRN-mediated activation of integrin α5β1 survival signaling protects dopaminergic neurons during cellular injury. Future α-synuclein aggregation or propagation models will be required to determine whether this pathway also affects α-synuclein accumulation, spreading, or α-synuclein-associated neurodegeneration.

Together with prior reports that integrin α5β1 regulates neuronal adhesion dynamics and trophic signaling [19,24,50], our results suggest that PHSRN’s neuroprotection arises from restoring a critical survival pathway that becomes compromised in PD-relevant neuronal damage. Mechanistically, PHSRN activates integrin α5β1 signaling to restore pro-survival kinase pathways that are compromised during neurotoxic stress. Rotenone markedly reduced phosphorylation of FAK, PI3K, Akt, and ERK, indicating suppression of integrin-linked survival signaling; PHSRN treatment reinstated phosphorylation of all four kinases. Reactivation of the FAK–PI3K–Akt and ERK cascades provides a clear molecular basis for the reduced neuronal apoptosis observed with PHSRN, as these pathways converge on anti-apoptotic effectors. Consistent with this, PHSRN significantly diminished rotenone-induced cleaved caspase-3 and -8, demonstrating inhibition of both intrinsic and extrinsic apoptotic signaling. Moreover, Akt activation is known to facilitate Nrf2/ARE antioxidant responses [51,52], and PHSRN correspondingly reduced ROS accumulation while preserving glutathione redox balance (GSH/GSSG), indicating enhanced intrinsic oxidative defense. These data support a model in which integrin α5β1 activation by PHSRN stabilizes neuronal integrity by simultaneously sustaining pro-survival signaling, suppressing apoptotic execution, and strengthening antioxidant capacity under mitochondrial stress.

Furthermore, integrin α5β1 signaling intersects with neurotrophin regulatory pathways. Prior studies show that integrin-binding peptides such as soluble RGD can increase BDNF and NGF expression in hippocampal neurons through integrin-dependent Ca^2+^ influx [53,54], indicating that integrin activation can directly enhance trophic support. Consistent with this, we observed that PHSRN restored BDNF and NGF levels in MPTP-lesioned mice, supporting the view that integrin α5β1 engagement promotes neurotrophic factor expression under neurodegenerative stress. This concept is reinforced by evidence from stroke models in which PHSRN activates the integrin α5–FAK–ERK axis to stimulate VEGF release and angiogenesis [24,55,56], demonstrating that integrin α5 activation can broadly induce reparative and growth-promoting responses in the injured brain. Taken together, our data indicate that PHSRN-triggered α5β1 activation initiates a coordinated neuroprotective program that includes restoration of pro-survival kinase signaling, suppression of oxidative injury via Nrf2-associated defenses, upregulation of neurotrophic factors, and preservation of synaptic and dopaminergic neuron integrity.

Importantly, our data demonstrate that PHSRN’s neuroprotective effects require integrin α5β1 engagement. Knockdown of integrin α5 eliminated PHSRN’s neuroprotective effect. Without the receptor, PHSRN no longer prevented neurite loss or dopaminergic degeneration, demonstrating that its efficacy is integrin α5-dependent. In vivo, targeted integrin α5 knockdown within the nigrostriatal pathway similarly aggravated MPTP-induced motor deficits and dopaminergic neuron loss, reinforcing the essential role of α5β1 signaling in the intact system. Integrin α5β1 is known to support neuronal survival and synaptic maintenance [19,24,57,58]; our findings extend this function to the context of parkinsonian stress by showing that α5β1 activity serves as an endogenous defense mechanism in dopaminergic neurons. Moreover, the loss of PHSRN benefit after α5 knockdown mirrors prior evidence that disrupting α5β1 signaling can worsen outcomes in CNS injury models [19,59]. Together, these results provide direct mechanistic evidence that PHSRN confers neuroprotection through integrin α5β1, identifying this receptor as both necessary and therapeutically targetable in PD-related neurodegeneration.

From a translational standpoint, Ac-PHSRN-NH_2_ has strong potential as a disease-modifying therapy for Parkinson’s disease. Current treatments such as L-DOPA and deep brain stimulation primarily alleviate symptoms without altering the trajectory of dopaminergic neurodegeneration [4,60]. In contrast, PHSRN activates integrin α5β1-dependent survival and repair pathways, which in our models were associated with preserved dopaminergic neuron viability, synaptic connectivity, and improved motor behavior. This suggests that PHSRN may complement existing symptomatic treatments by influencing processes related to neuronal maintenance rather than solely compensating for dopamine loss. In addition, PHSRN showed efficacy when administered systemically. Although small peptides often face stability and delivery challenges, the acetylated PHSRN motif demonstrated sufficient activity to elicit central effects in vivo under the conditions tested. Previous studies in stroke models have similarly reported that peripherally delivered PHSRN can influence brain regions undergoing injury [24,32], supporting the possibility that this peptide can act within the central nervous system without invasive administration. Moreover, other fibronectin-mimetic integrin ligands have progressed into clinical evaluation for non-neurological indications [61,62,63,64], indicating that the general therapeutic platform is feasible, though its applicability to chronic neurodegenerative disease remains to be fully defined. Future studies should assess long-term safety, refine dosing and delivery, and evaluate efficacy in chronic and α-synuclein-based PD models. Our findings provide proof-of-concept that targeting integrin α5β1 protects dopaminergic neurons from degeneration. By preserving dopamine production, synaptic structure, and motor function, Ac-PHSRN-NH_2_ shows potential as a disease-modifying therapy rather than a purely symptomatic treatment.

A limitation of this study is that MPTP metabolism and MPP^+^ levels were not directly measured after PHSRN treatment, so possible effects on MPTP conversion, distribution, or clearance cannot be excluded. Nevertheless, the rotenone cell model, which bypasses MPTP-to-MPP^+^ conversion and directly inhibits mitochondrial complex I, showed similar PHSRN-mediated cytoprotection. This supports an integrin α5β1-dependent mitochondrial and survival mechanism, although future measurement of striatal and midbrain MPP^+^ levels will be needed for further validation. In addition, the limited reduction in dopamine levels shown in Figure 9B should be interpreted in the context of the bulk midbrain tissue sampling approach. Although the tissue dissections were targeted to the SNc-containing midbrain region, inclusion of adjacent or less affected midbrain structures may have diluted the apparent dopamine loss. Therefore, dopamine depletion within the SNc may have been underestimated. Future TH immunostaining with region-specific quantification of the SNc, VTA, and surrounding midbrain regions will help clarify the anatomical extent of dopaminergic neuronal loss.

Although PHSRN showed neuroprotective effects in the present PD model, the mechanism by which systemically administered PHSRN accesses the injured brain remains unclear. We did not directly measure PHSRN penetration across the BBB in this study. In our previous ischemic stroke study, however, PHSRN was shown to target the damaged brain region and distribute to endothelial cells after intraperitoneal administration, suggesting that PHSRN can reach injured CNS regions under pathological vascular conditions [24]. In PD, BBB dysfunction has been increasingly recognized and may involve tight-junction disruption, altered endothelial transport, vascular degeneration, neuroinflammation, and immune-cell infiltration [65]. Consistently, MPTP treatment has been reported to induce BBB leakage and reduce tight-junction proteins, including occludin and ZO-1, particularly in the striatum [66]. Therefore, BBB impairment in the PD brain may partially facilitate PHSRN access to the injured nigrostriatal system. Further studies using labeled PHSRN, pharmacokinetic tracking, and BBB permeability assays are needed to define the precise route and extent of PHSRN brain entry.

## 5. Conclusions

Our findings suggest that activation of integrin α5β1 by PHSRN demonstrates a potential neuroprotective approach in Parkinson’s disease. PHSRN restores pro-survival signaling, enhances antioxidant capacity, and helps preserve dopaminergic neuronal and synaptic integrity, thereby counteracting key mechanisms of neurodegeneration. These results support the possibility that integrin-targeted strategies could contribute to slowing disease progression, complementing current symptomatic treatments.

## Data Availability

All data supporting the findings of this study are presented within the article (Figure 1, Figure 2, Figure 3, Figure 4, Figure 5, Figure 6, Figure 7, Figure 8 and Figure 9). Additional data relevant to this work are available from the corresponding author (C.C. Wu) upon reasonable request.

## Supplementary Materials

The following supporting information can be downloaded at: https://www.mdpi.com/article/10.3390/antiox15070822/s1, Figure S1. Validation of integrin α5 knockdown efficiency in vivo and in vitro.

## Abbreviations

AKT, protein kinase B; ANOVA, analysis of variance; BDNF, brain-derived neurotrophic factor; CM, conditioned medium; DA, dopamine; DAPI, 4′,6-diamidino-2-phenylindole; ECM, extracellular matrix; ELISA, enzyme-linked immunosorbent assay; ERK, extracellular signal–regulated kinase; FAK, focal adhesion kinase; GPX4, glutathione peroxidase 4; GSH, reduced glutathione; GSSG, oxidized glutathione; GSTP1, glutathione S-transferase Pi 1; HTS, high-throughput screening; IHC, immunohistochemistry; IF, immunofluorescence; KD, knockdown; LDH, lactate dehydrogenase; MPTP, 1-methyl-4-phenyl-1,2,3,6-tetrahydropyridine; NGF, nerve growth factor; NRF2, nuclear factor erythroid 2–related factor 2; OCT, optimal cutting temperature compound; PBS, phosphate-buffered saline; PCR, polymerase chain reaction; PD, Parkinson’s disease; PFA, paraformaldehyde; PI3K, phosphoinositide 3-kinase; RIPA, radioimmunoprecipitation assay buffer; ROS, reactive oxygen species; SD, standard deviation; shRNA, short hairpin RNA; SNc, substantia nigra pars compacta; SOD1, superoxide dismutase 1; TH, tyrosine hydroxylase; TUNEL, terminal deoxynucleotidyl transferase dUTP nick-end labeling; WB, Western blot.
