# Supplementary material for: Integrin α5β1 Activation by PHSRN Peptide Elicits Neuroprotection and Functional Recovery in Parkinson’s Disease Mice
Source: Antioxidants (Basel). 2026 Jun 30;15(7):822. doi: 10.3390/antiox15070822 (PMC13405216; doi:10.3390/antiox15070822)
Supplement: Supplementary file 1 [file antioxidants-15-00822-s001.zip › antioxidants-4328899-supplementary.pdf]

Supporting Information

Integrin α5β1 Activation by PHSRN Peptide Elicits Neuroprotection and Functional Recovery in Parkinson's Disease Mice

Cheng-Chun Wu [1,2,†], Hao-Kuang Wang [1,3,†], Yu-Ting Su [4], Yu-Cheng Ho [2], Yuan-Chin Hsieh [5], Cheng-Loong Liang [2,3], Yung-Kuo Lee [6,7,8], Tian-Huei Chu [6], Yun-Shin Lin [9] and Jui-Sheng Chen [1,3,*]

[1] Graduate Institute of Medicine, College of Medicine, I-Shou University, Kaohsiung City 824005, Taiwan; chengchunwu@isu.edu.tw (C.-C.W.); ed101393@gmail.com (H.-K.W.)
[2] School of Medicine, College of Medicine, I-Shou University, Kaohsiung City 824005, Taiwan; ycho@isu.edu.tw (Y.-C.H.); p0201@edah.org.tw (C.-L.L.)
[3] Department of Neurosurgery, E-DA Hospital, I-Shou University, Kaohsiung City 824005, Taiwan
[4] Department of Obstetrics and Gynecology, Kaohsiung Chang Gung Memorial Hospital and Chang Gung University College of Medicine, Kaohsiung City 833401, Taiwan; kimyy9487@cgmh.org.tw
[5] Department of Occupational Therapy, College of Medicine, I-Shou University, Kaohsiung City 824005, Taiwan; hyc1014@isu.edu.tw
[6] Medical Laboratory, Medical Education and Research Center, Kaohsiung Armed Forces General Hospital, Kaohsiung City 802231, Taiwan; yungkuolee@gmail.com (Y.-K.L.); skbboyz0817@gmail.com (T.-H.C.)
[7] Division of Experimental Surgery Center, Department of Surgery, Tri-Service General Hospital, National Defense Medical University, Taipei 114201, Taiwan
[8] School of Medicine, National Defense Medical University, Taipei 114201, Taiwan
[9] Department of Psychiatry, Kaohsiung Armed Forces General Hospital, Kaohsiung City 802231, Taiwan; mysing@gmail.com
* Correspondence: dumboschen@gmail.com; Tel.: +886-7-6151100-7960; Fax: +886-7-6155150
† These authors contributed equally to this work.

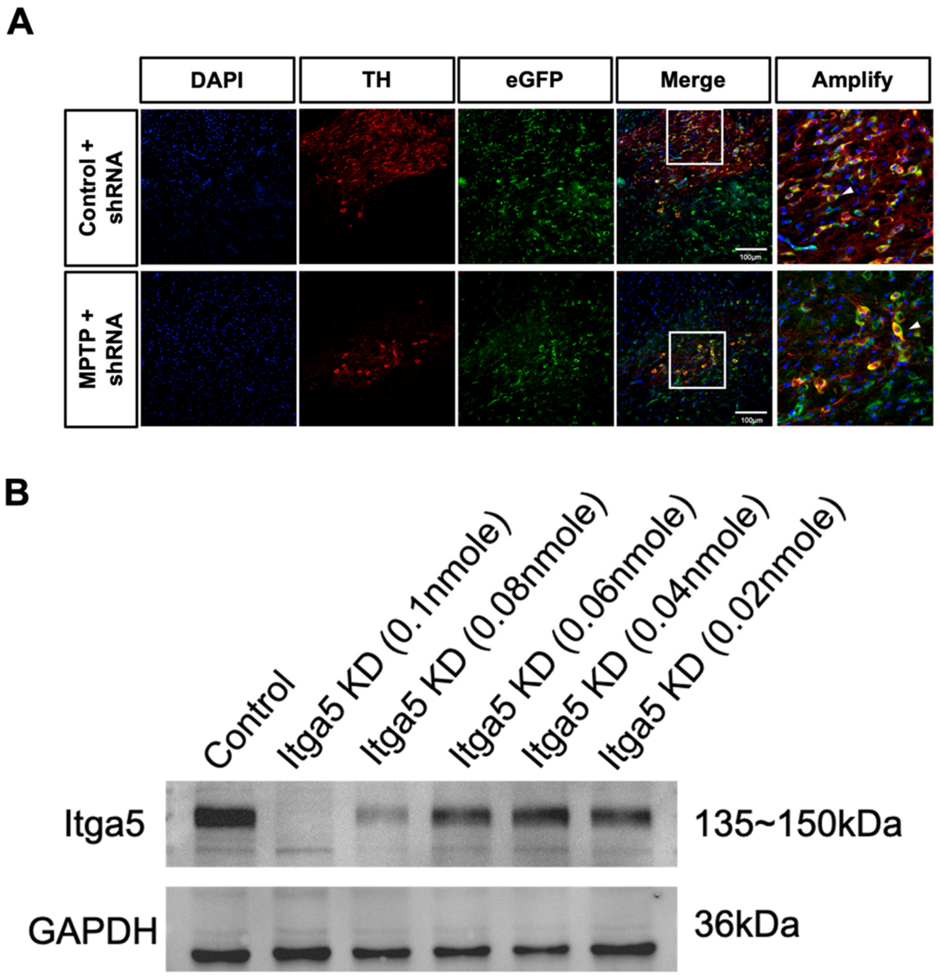

Figure S1. Validation of integrin α5 knockdown efficiency in vivo and in vitro.

(A) Representative immunofluorescence images of the substantia nigra pars compacta (SNc) showing tyrosine hydroxylase (TH, red), eGFP (green), and DAPI (blue) staining in Control + shRNA and MPTP + shRNA mice. Merged and magnified views demonstrate successful lentiviral delivery and regional expression of the shRNA construct. Scale bar = 50 µm.

(B) Western blot analysis confirming dose-dependent knockdown efficiency of integrin α5 (Itga5) in N27 dopaminergic neurons transduced with increasing amounts of Itga5 shRNA lentivirus (0.02–0.1 nmole). GAPDH served as the loading control.
